# A real-world observational study of second-line anti-TNFα treatment in patients with ulcerative colitis who received vedolizumab as a first-line biologic

**DOI:** 10.1093/crocol/otag042

**Published:** 2026-05-15

**Authors:** Chiahung Chou, Marie Sanchirico, Precious A Anyanwu, Lucinda J Van Anglen, Timothy E Ritter

**Affiliations:** Takeda Pharmaceuticals USA, Cambridge, MA 02142, United States; Takeda Pharmaceuticals USA, Cambridge, MA 02142, United States; Healix Infusion Therapy, LLC, Sugar Land, TX 77479, United States; Healix Infusion Therapy, LLC, Sugar Land, TX 77479, United States; GI Alliance, Southlake, TX 76092, United States

**Keywords:** anti-tumor necrosis factor α, real-world data, ulcerative colitis, vedolizumab

## Abstract

**Background:**

Many patients with ulcerative colitis (UC) require treatment with more than one biologic during their lifetime. This real-world observational study assessed the effectiveness of second-line anti-tumor necrosis factor α (anti-TNFα) treatment in patients with UC who had previously received vedolizumab as a first-line biologic.

**Methods:**

This retrospective study included biologic-naive adult patients with UC who were treated with vedolizumab as a first-line biologic at a large, multicenter, private gastroenterology practice in Texas, United States, between January 1, 2018, and December 31, 2020. Outcomes were assessed by partial Mayo score, not including endoscopic data.

**Results:**

In total, 260 patients received vedolizumab. Treatment was discontinued in 53 patients who then received a second-line anti-TNFα treatment. Of all patients treated with a second-line anti-TNFα treatment, 14/53 (26.4%) had a clinical response and 11/53 (20.8%) had clinical remission at 12 months. Accounting for anti-TNFα treatment discontinuations, 14/26 patients (53.8%) had a clinical response, and 11/26 patients (42.3%) had clinical remission at 12 months, respectively. In an exploratory analysis of the patients who received vedolizumab as a first-line biologic, 188/260 (72.3%) remained on treatment at 12 months and 149/188 (79.3%) of them had clinical remission. For those with a partial Mayo score ≥ 5 at baseline, onset of vedolizumab action was observed in 49.6% by week 2 and in 87.2% by week 6.

**Conclusions:**

Second-line anti-TNFα treatments were effective following vedolizumab as a first-line biologic. Most patients had onset of action of vedolizumab within 6 weeks and had clinical remission at 12 months.

## Graphical Abstract

**Graphical Abstract:**
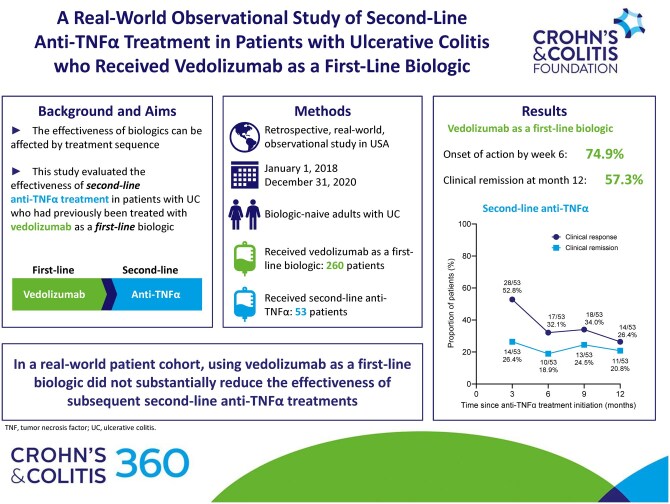

## Introduction

Ulcerative colitis (UC) is a progressive disease characterized by chronic inflammation of the colon and rectum that can result in irreversible damage to the bowel.^1^^,^^2^ When not optimally managed, UC can result in anatomical and functional abnormalities, extraintestinal manifestations, and the need for surgery.^2^ These factors can lead to long-term disability and impaired quality of life for people living with UC. Disease management strategies in UC are moving toward earlier intervention with biologic drugs to try to modify disease progression and prevent long-term bowel damage.^3^

In clinical trials, biologics have been shown to be effective in treating patients with moderate to severe UC.^4–6^ Although anti-TNFα treatments were the first biologics to be approved and remain commonly used as first-line biologic treatments, recent direct comparative evidence suggests that vedolizumab may be more clinically effective as a first-line biologic than anti-TNFα treatments in people with UC.^7–10^ For example, in the prospective head-to-head VARSITY study, clinical remission at week 52 in biologic-naive patients was observed in 34.2% of patients who received vedolizumab as a first-line biologic and 24.3% who received first-line adalimumab.^8^

Although biologic drugs are effective and can sustain remission over long periods in many patients, some patients do not experience an adequate response to first-line biologic treatment or lose their initial positive response to treatment over time. In this instance, patients may need to switch to a second-line biologic.^11^^,^^12^ In some studies, the clinical effectiveness of vedolizumab has been demonstrated to be lower for patients who have received a prior anti-TNFα treatment than for those who are biologic-naive.^8^^,^^13^ In a post hoc analysis of data from the GEMINI 1 study, clinical remission at week 52 was observed in 46.9% of biologic-naive patients who received vedolizumab as a first-line biologic compared with 36.1% who had previously received an anti-TNFα treatment.^13^ In the VARSITY study, the absolute difference in clinical remission at week 52 between patients who received vedolizumab and those who received adalimumab was greater in biologic-naive patients (9.9%) than those who had received a prior anti-TNFα treatment (4.2%).^8^ In a meta-analysis, being biologic-naive was associated with a higher probability of clinical remission at week 52 in patients with UC (relative risk, 1.32; 95% CI, 1.14-1.53).^14^ It is important to note, however, that other studies have shown that the effectiveness of vedolizumab as a second-line advanced therapy in patients who previously received anti-TNFα treatment remains comparable with other advanced therapies in biologic-exposed patients.^15–17^ Nonetheless, to maximize the impact of both first-line and potential second-line therapies, the choice of first-line biologic should not only be influenced by comparative effectiveness but also by the impact that treatment sequence has on the effectiveness of second-line treatments.

The effectiveness of anti-TNFα treatments when they are used as second-line treatments following vedolizumab as a first-line biologic remains uncertain, and available data are sparse. In patients with UC in the retrospective, multicenter EVOLVE study, clinical remission rates 3 and 6 months after initiating first-line anti-TNFα treatment (9.7% and 19.6%, respectively) were similar to rates 3 and 6 months after initiating a second-line anti-TNFα treatment (11.0% and 14.7%, respectively) following vedolizumab as the first-line biologic.^18^ However, the cohorts in this study were too small to draw definitive conclusions.

Previously, we reported data from a limited number of patients that also suggested that the effectiveness of anti-TNFα treatments was not reduced when they were used in the second line following vedolizumab.^19^ The objective of this study was to provide further real-world data on the use of second-line anti-TNFα treatments following vedolizumab to shed light on the optimal biologic treatment sequence for patients with UC.

## Materials and methods

### Study design and patient population

This single-cohort, noncomparative, retrospective, observational study included patients aged 18 years or older who had a diagnosis of UC and were treated at a large, multicenter, private gastroenterology practice in Texas, United States, with over 80 locations and over 400 healthcare providers. Eligible patients were biologic-naive before first receiving vedolizumab as a first-line biologic treatment during the enrollment period, which began on January 1, 2018, and ended on December 31, 2020. The study index period was from January 1, 2018, to December 31, 2021, to allow up to 12 months of follow-up for the last enrolled patient. The index date for each patient was the date of their first vedolizumab infusion. No prespecified decision criteria were used to determine which patients received vedolizumab as a first-line biologic. The choice to provide vedolizumab to any individual patient was made by the healthcare provider based upon their experience and clinical data available to them at the time.

Patients who discontinued first-line vedolizumab were required to have switched to a second-line anti-TNFα treatment and have 12 months of follow-up data after switching unless censored for discontinuation of the second-line anti-TNFα treatment, transfer of care, or death. Patients who remained on first-line vedolizumab were required to have 12 months of follow-up data after the index date. Patients who had prior exposure to any biologic or who had a diagnosis of Crohn’s disease were excluded.

### Outcomes

The primary outcome was the proportion of patients who discontinued first-line vedolizumab and had a clinical response (defined as ≥ 2-point reduction in partial Mayo [pMayo] score from baseline) to second-line anti-TNFα treatment at 3, 6, 9, and 12 months after initiation of second-line treatment.

Secondary outcomes included the proportion of all patients who received first-line vedolizumab who switched to a second-line anti-TNFα treatment and the proportion of patients who switched to second-line anti-TNFα treatment who had clinical remission (defined as a pMayo score < 2) at 3, 6, 9, and 12 months after initiation of second-line treatment. For calculation of the proportion of patients with corticosteroid-free remission, the denominator was patients who were receiving an anti-TNFα treatment at each time point and who had been receiving a corticosteroid at baseline.

Exploratory outcomes included: the proportion of patients who received first-line vedolizumab who had clinical remission at 3, 6, 9, and 12 months after initiation of first-line treatment; the proportion of patients who received first-line vedolizumab who had corticosteroid-free remission at 3, 6, 9, and 12 months after initiation of first-line treatment; and the proportion of patients who had onset of action of vedolizumab (defined as a decrease of ≥ 1 point in rectal bleeding score or ≥ 1 point in stool frequency score from baseline) at weeks 2 and 6 after initiation of first-line treatment.

### Data collection and statistical analysis

Patient data were collected by trained personnel from electronic medical records held by GI Alliance and Healix Infusion Therapy using a data collection instrument implemented in Microsoft Excel software. Baseline demographic information collected included patient age at the time of vedolizumab initiation, sex, smoking status, and Charlson Comorbidity Index score. Baseline disease and treatment characteristic information collected included time since UC diagnosis, concurrent use of nonbiologic treatments, UC disease history, reasons for vedolizumab discontinuation (if applicable), and laboratory data. Data quality and integrity were ensured through regular audits of data entry, cross-verification between electronic medical records and manual chart reviews, and the use of standardized data collection forms. Any discrepancies in data were resolved through additional chart reviews. The study team maintained strict protocols to minimize data entry errors and ensure consistent application of diagnostic criteria across all study sites.

Disease activity and treatment effectiveness (at baseline and at 3, 6, 9, and 12 months after treatment initiation) were assessed using the pMayo score comprising three non-endoscopic subscores: stool frequency score; rectal bleeding score; and the physician’s global assessment. pMayo scores of 2-4, 5-7, and > 7 signified mild, moderate, and severe disease activity, respectively. Patients with a pMayo score < 2 were in remission. Effectiveness of first-line vedolizumab and second-line anti-TNFα treatment are shown as a proportion of the intent-to-treat population (the denominator is the number of patients who initiated treatment with each drug) and the population remaining on treatment at each assessment timepoint (the denominator is number of patients that remain on each treatment at 3, 6, 9, and 12 months, respectively).

Descriptive statistics for continuous variables are reported as means and SDs or medians and interquartile ranges (IQRs). Categorical variables are reported as percentages. No statistical testing was performed to compare differences in baseline demographics and disease characteristics between groups. Cumulative time to first clinical response and to clinical remission were estimated using the Kaplan-Meier method. Multivariate analyses were two-tailed with an α value of 0.05 and were conducted in the entire cohort to determine factors associated with clinical remission and treatment persistence with vedolizumab at 12 months. All statistical analyses were conducted using SAS 9.4 (SAS Institute Inc., NC, USA).

### Ethics

The institutional review board was provided by the Biomedical Research Alliance of New York Institutional Review Board on January 27, 2023. The institutional review board protocol number for the study is 23-08-446-1297. The study was conducted in accordance with the protocol, the International Society for Pharmacoepidemiology Guidelines for Good Pharmacoepidemiology Practices, all applicable state and federal institutional review board regulations, all applicable local regulations, and ethical principles originating from the Declaration of Helsinki. All authors had access to the study data and reviewed and approved the final manuscript.

## Results

### Baseline demographics and disease characteristics

In total, 260 patients received vedolizumab as a first-line biologic treatment during the index period. The baseline demographics and disease characteristics of patients who received vedolizumab as a first-line biologic and those who discontinued vedolizumab and subsequently received a second-line anti-TNFα treatment (*n* = 53) are shown in Table 1. The mean (SD) age of those who received vedolizumab as a first-line biologic treatment (42.6 years [15.7]) was slightly lower than that of patients who discontinued treatment and received a second-line anti-TNFα treatment (44.3 years [16.0]). Evaluation of pMayo scores revealed that some enrolled patients had mild disease (pMayo score 2-4) or were in remission (pMayo score < 2) at baseline. Among all patients who received vedolizumab as a first-line biologic and those who received a second-line anti-TNFα treatment, 45.0% and 52.8%, respectively, were female.

**Table 1:** Baseline demographic and disease characteristics.

	First-line vedolizumab^a^	Second-line anti-TNFα^b^
Demographic or disease characteristic	*N* = 260	*n* = 53
**Age, y, mean (SD)**	42.6 (15.7)	44.3 (16.0)
**Age category, y, n (%)**		
** 18-34**	90 (34.6)	18 (34.0)
** 35-64**	148 (56.9)	31 (58.5)
** ≥ 65**	22 (8.5)	4 (7.5)
**Sex, n (%)**		
**Male**	143 (55.0)	25 (47.2)
**Female**	117 (45.0)	28 (52.8)
**Smoking status, n (%)**		
** Never smoked**	192 (73.8)	34 (64.2)
** Former smoker**	54 (20.8)	16 (30.2)
** Current smoker**	14 (5.4)	3 (5.7)
**Baseline partial Mayo score, n (%)**		
** < 2**	41 (15.8)	5 (9.4)
** 2-4**	96 (36.9)	13 (24.5)
** 5-7**	84 (32.3)	21 (39.6)
** >7**	39 (15.0)	14 (26.4)
**Other characteristics, median (IQR)**		
**Body mass index, kg/m^2^**	25.9 (22.1-29.4)	25.2 (22.2-32.2)
**Charlson Comorbidity Index score**	0 (0-1)	0 (0-2)
**Time from diagnosis to receiving vedolizumab, y**	3.2 (0.9-9.1)	3.8 (1.0-9.4)
**Time from vedolizumab referral to induction, days**	32.0 (18.0-48.0)	18.0 (2.0-32.0)
**Duration of vedolizumab treatment during 12-month follow-up, months**	12.0 (9.6-12.0)	7.5 (5.0-12.0)

Abbreviations: IQR, interquartile range; TNF, tumor necrosis factor.

a Patients were biologic-naive and received vedolizumab as a first-line biologic treatment but may have received prior conventional therapy.

b Patients who received vedolizumab as a first-line biologic before switching to a second-line anti-TNFα treatment.

**Figure 1:**
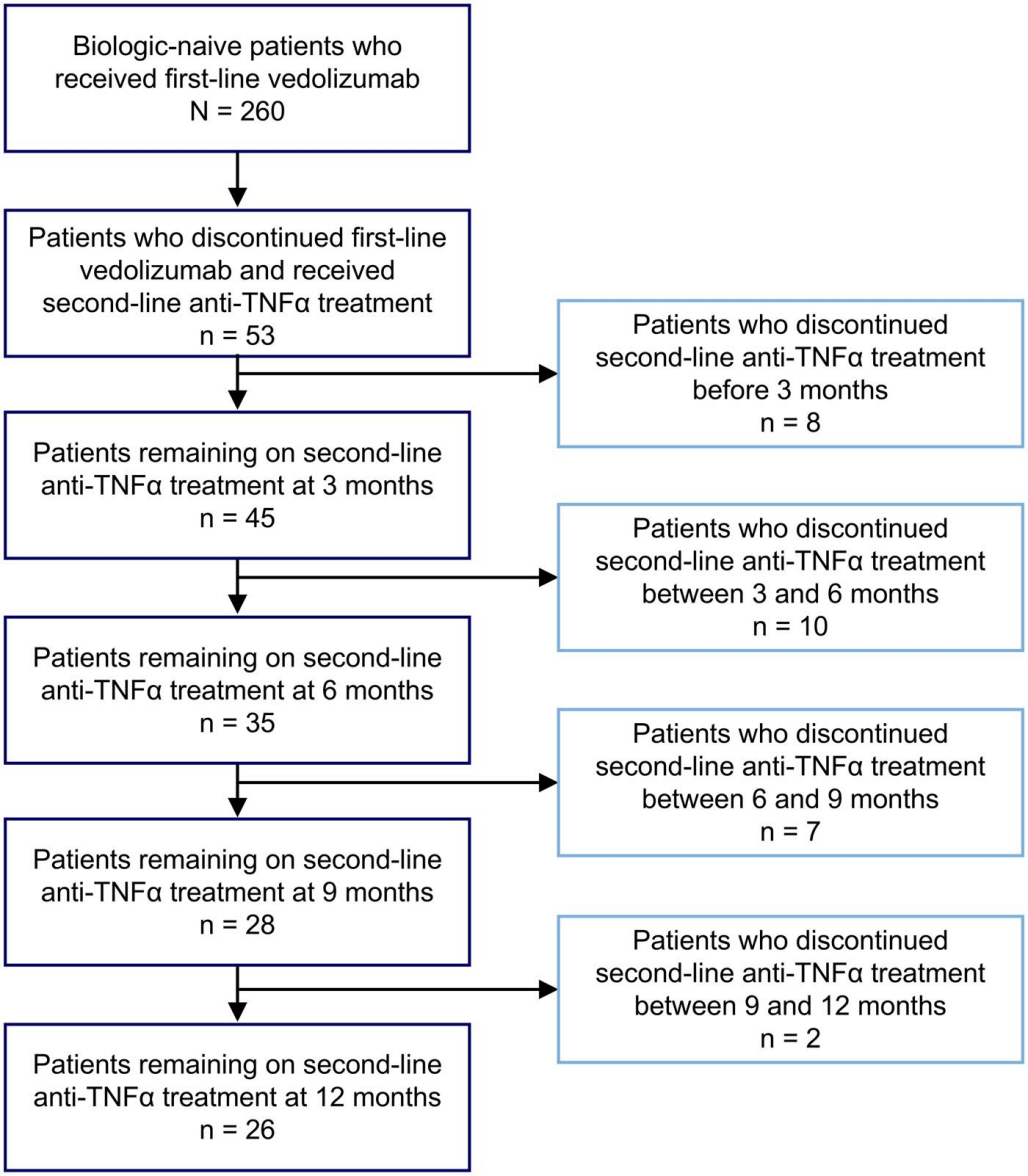
Patient disposition. TNF, tumor necrosis factor.

In patients who discontinued vedolizumab and received a second-line anti-TNFα treatment, the median (IQR) time between diagnosis and the initiation of vedolizumab as a first-line biologic was 3.8 years (1.0-9.4) compared with 3.2 years (0.9-9.1) in the overall group who received vedolizumab; before receiving vedolizumab as a first-line biologic, all patients were treated with conventional UC therapies. Of all patients who received vedolizumab, 72 (27.7%) discontinued treatment within 12 months of initiation. Secondary loss of response to vedolizumab was the most frequent reason for first-line treatment discontinuation (Table 2). Of the patients who discontinued first-line treatment, 53 patients received an anti-TNFα treatment and had 12 months of follow-up data after initiating second-line treatment. The second-line anti-TNFα treatments received following vedolizumab discontinuation were infliximab (*n* = 39, 73.6%) and adalimumab (*n* = 14, 26.4%). Twelve months after beginning an anti-TNFα treatment, 26 patients (49.1%) remained on second-line treatment, and 27 patients (50.9%) had discontinued treatment (Figure 1).

**Table 2:** Baseline treatment characteristics for vedolizumab as a first-line biologic and second-line anti-TNFα treatments.

	Patients who received first-line vedolizumab		Patients who received second-line anti-TNFα treatment	
Treatment characteristic	First-line vedolizumab**^a^**	Second-line anti-TNFα^b^	Infliximab	Adalimumab
**Patients, n**	260	53	39	14
**Dose, median (IQR)**	300 mg	300 mg	5.2 (5.0-5.7) mg/kg	40 mg
**Initial dose frequency**	Q8W	Q8W	Q8W	Q2W
**Dose escalation, n (%)**				
** Increased dose**	0 (0.0)	0 (0.0)	11 (28.2)	0 (0.0)
** Increased frequency**	40 (15.4)	27 (50.9)	3 (0.1)	4 (28.6)
** Discontinued vedolizumab after dose escalation**	28 (10.8)	27 (50.9)	–	–
**Concurrent medication,^c^ n (%)**				
** 5-ASA only**	74 (28.5)	14 (26.4)	12 (30.8)	2 (14.3)
** Immunomodulator only**	7 (2.7)	0 (0.0)	0 (0.0)	0 (0.0)
** 5-ASA + immunomodulator**	5 (1.9)	0 (0.0)	0 (0.0)	0 (0.0)
** Corticosteroid only**	52 (20.0)	8 (15.1)	6 (15.4)	2 (14.3)
** Corticosteroid + 5-ASA**	95 (36.5)	24 (45.3)	15 (38.5)	9 (64.3)
** Corticosteroid + immunomodulator**	4 (1.5)	0 (0.0)	0 (0.0)	0 (0.0)
** Corticosteroid + 5-ASA + immunomodulator**	7 (2.7)	3 (5.7)	2 (5.1)	1 (7.1)
** Any corticosteroid^d^**	158 (60.8)	35 (66.0)	23 (59.0)	12 (85.7)
**Discontinuation characteristics**				
** Discontinued vedolizumab, n (%)**	72 (27.7)	53 (100)	–	–
** Time to discontinuation, months, median (IQR)**	6.0 (3.3-10.8)	7.5 (5.0-14.0)	–	–
**Reason for discontinuation^e^**				
** Primary nonresponse**	5 (6.9)	3 (5.7)	–	–
** Secondary loss of response**	29 (40.3)	41 (77.4)	–	–
** Transfer of care**	13 (18.1)	0 (0.0)	–	–
** Lost to follow-up**	9 (12.5)	0 (0.0)	–	–
** Competing medical problem**	6 (8.3)	0 (0.0)	–	–

Abbreviations: 5-ASA, 5-aminosalicylic acid; IQR, interquartile range; Q2W, every 2 weeks; Q8W, every 8 weeks; TNF, tumor necrosis factor.

a Vedolizumab treatment characteristics in all patients who were biologic-naive and received vedolizumab as a first-line biologic treatment.

b Vedolizumab treatment characteristics in patients who received vedolizumab as a first-line biologic before switching to a second-line anti-TNFα treatment.

c Concurrent medications being used at the time of initiation of first-line vedolizumab or second-line anti-TNFα treatment.

d Any patients who were receiving an oral corticosteroid alone or in combination with 5-ASA or an immunomodulator.

e Patients who discontinued first-line vedolizumab; only categories that included more than five patients are shown.

At baseline, among all patients who received vedolizumab as a first-line biologic, 158 patients (60.8%) were receiving a corticosteroid either alone or in combination with 5-aminosalicylic acid and/or an immunomodulator. Of those who subsequently received a second-line anti-TNFα treatment, 35 patients (66.0%) were receiving a corticosteroid at the time of initiating vedolizumab (Table 2).

### Effectiveness of second-line anti-TNFα treatment

**Figure 2:**
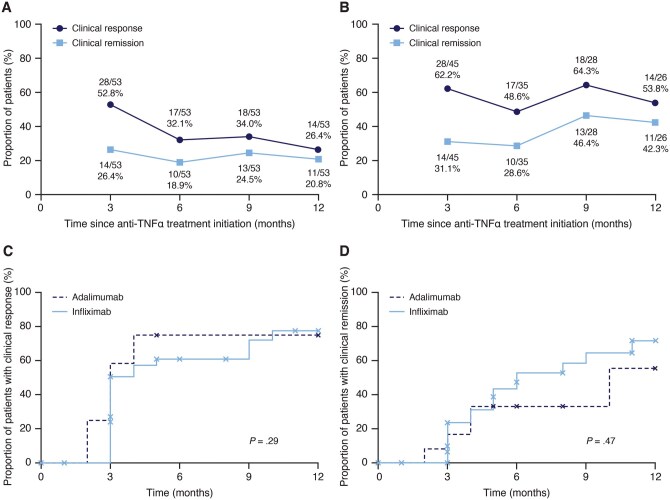
Clinical response and clinical remission with second-line anti-TNFα treatment for (A) all patients who received a second-line anti-TNFα treatment after previously receiving vedolizumab as a first-line biologic (intent to treat population) and (B) patients who remained on anti-TNFα treatment at each assessment time point. Cumulative time to first clinical response (C) and first clinical remission (D) with second-line anti-TNFα treatment. TNF, tumor necrosis factor.

The proportion of patients who had a clinical response (primary outcome; ≥ 2-point reduction in pMayo score from baseline) and the proportion who had clinical remission (secondary outcome; a pMayo score < 2) at 3, 6, 9, and 12 months after treatment initiation among all patients who received a second-line anti-TNFα treatment (intent-to-treat population; *n* = 53) are shown in Figure 2A. Three months after beginning second-line anti-TNFα treatment, 28 patients (52.8%) had a clinical response, and 14 patients (26.4%) had clinical remission in the intent-to-treat population. By 12 months, the proportions of those who had a clinical response and clinical remission were 26.4% and 20.8%, respectively. Accounting for those who discontinued second-line anti-TNFα treatment before each assessment time point, at 3 months, of those who remained on anti-TNFα treatment, 62.2% had a clinical response, and 31.1% had clinical remission. At 12 months, the proportions were 53.8% and 42.3%, respectively (Figure 2B). Among patients who were receiving a corticosteroid at baseline and received a second-line anti-TNFα treatment, 5/31 patients (16.1%), 4/26 patients (15.4%), 5/17 patients (29.4%), and 5/15 patients (33.3%) had corticosteroid-free clinical remission at 3, 6, 9, and 12 months after initiation of second-line treatment, respectively. Although the numbers in each group may not be sufficient to draw conclusions about differences among anti-TNFα treatments, the clinical response and remission rates in the intent-to-treat population and the discontinuation population are shown for infliximab and adalimumab, respectively, in Figure S1.

In patients who received a second-line anti-TNFα treatment, the cumulative times taken to first clinical response and first clinical remission were similar for infliximab and adalimumab (Figure 2C and 2D). There was no significant difference between the two anti-TNFα treatments.

### Effectiveness of vedolizumab as a first-line biologic

**Figure 3:**
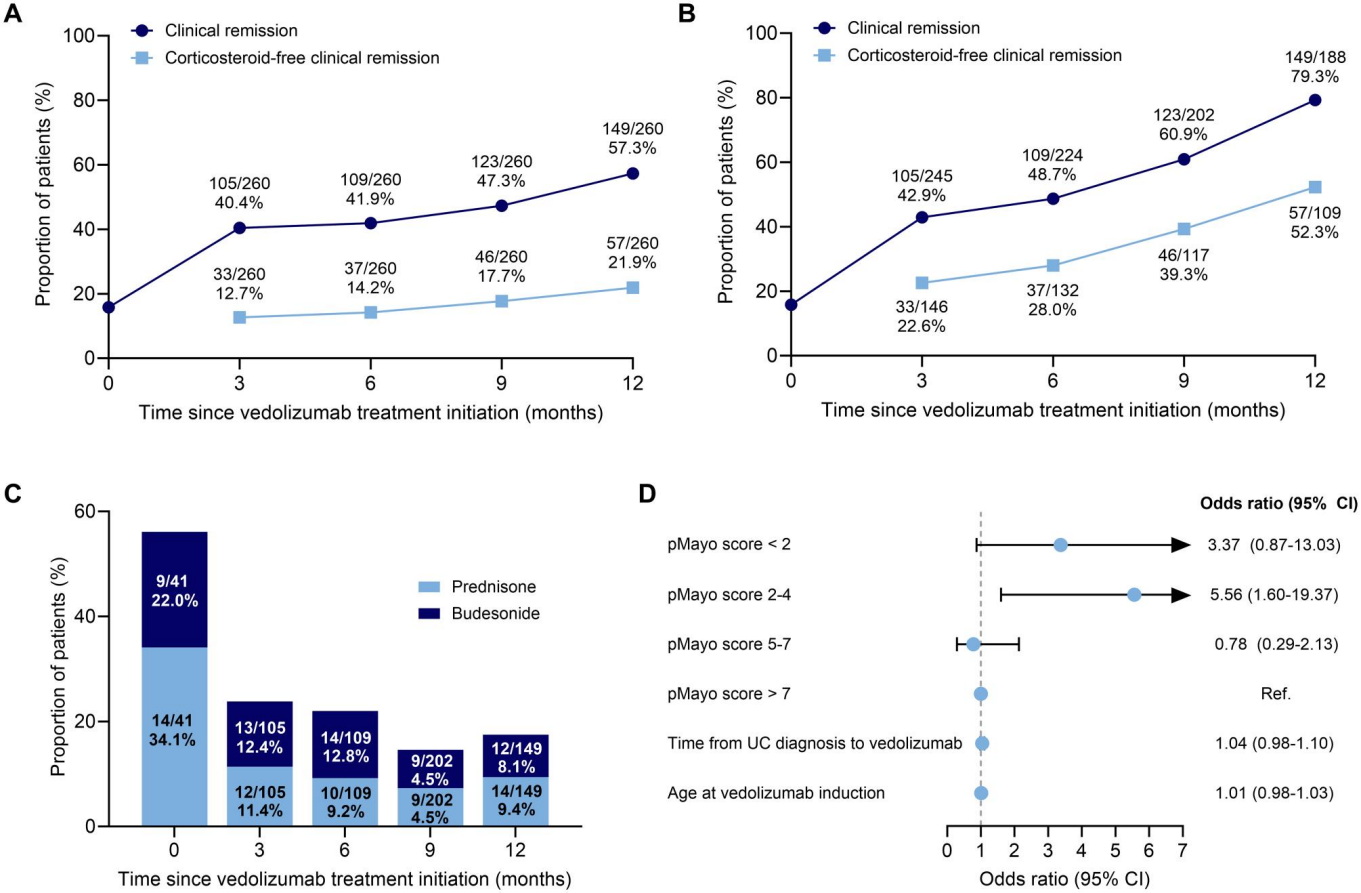
Clinical remission and corticosteroid-free clinical remission for first-line vedolizumab treatment for (A) all patients who received vedolizumab as a first-line biologic (intent to treat population) and (B) patients who remained on vedolizumab at each time point (the denominator for corticosteroid-free remission was patients who remained on vedolizumab at each time point and were receiving a corticosteroid at baseline). (C) Corticosteroid use among patients who had clinical remission at each time point. (D) Multivariate analysis of factors that influence the odds of having clinical remission at 12 months after treatment initiation; disease severity is measured at baseline. UC, ulcerative colitis.

In an exploratory analysis of the total cohort who received vedolizumab as a first-line biologic (intent-to-treat population; *n* = 260), the proportion of patients who had clinical remission and the proportion who had corticosteroid-free clinical remission at baseline and 3, 6, 9, and 12 months after treatment initiation for all patients who received vedolizumab as a first-line biologic are shown in Figure 3A. Three months after first-line biologic treatment initiation, 105 patients (40.4%) had clinical remission, and 33 patients (12.7%) had corticosteroid-free clinical remission. At 12 months, the proportions were 57.3% and 21.9%, respectively. Accounting for those who discontinued first-line biologic treatment with vedolizumab before each assessment time point, at 3 months, of those who remained on vedolizumab, 105/245 patients (42.9%) had clinical remission. At 12 months, 149/188 patients (79.3%) had clinical remission (Figure 3B).

Among patients who were receiving a corticosteroid at baseline and remained on vedolizumab but were no longer receiving a corticosteroid at 3 months (*n* = 146), 33 patients (22.6%) had clinical remission; at 12 months the proportion was 52.3% (Figure 3B). At baseline, 41/260 patients (15.8%) who received vedolizumab had clinical remission. Among these patients, 23/41 (56.1%) were receiving a concurrent corticosteroid; 34.1% were receiving prednisone and 22.0% were receiving budesonide (Figure 3C). At 3 months after vedolizumab treatment initiation, corticosteroid use in patients with clinical remission had decreased to 23.8% (*n* = 25/105). By 12 months after vedolizumab treatment initiation, corticosteroid use in those who had clinical remission was slightly lower than at 3 months (*n* = 26/149 [17.4%]) (Figure 3C).

Results of a multivariate analysis of factors that influence the odds of having clinical remission 12 months after initiating first-line biologic treatment with vedolizumab are shown in Figure 3D. Having a pMayo score of < 2 (remission) or 2-4 (mild disease) at baseline had odds ratios of 3.37 and 5.56, respectively, for clinical remission at 12 months. Compared with the reference group (pMayo score >7), the time from diagnosis to first vedolizumab infusion and the patient age at first vedolizumab infusion did not affect the odds of having clinical remission at 12 months.

### Vedolizumab onset of action

**Figure 4:**
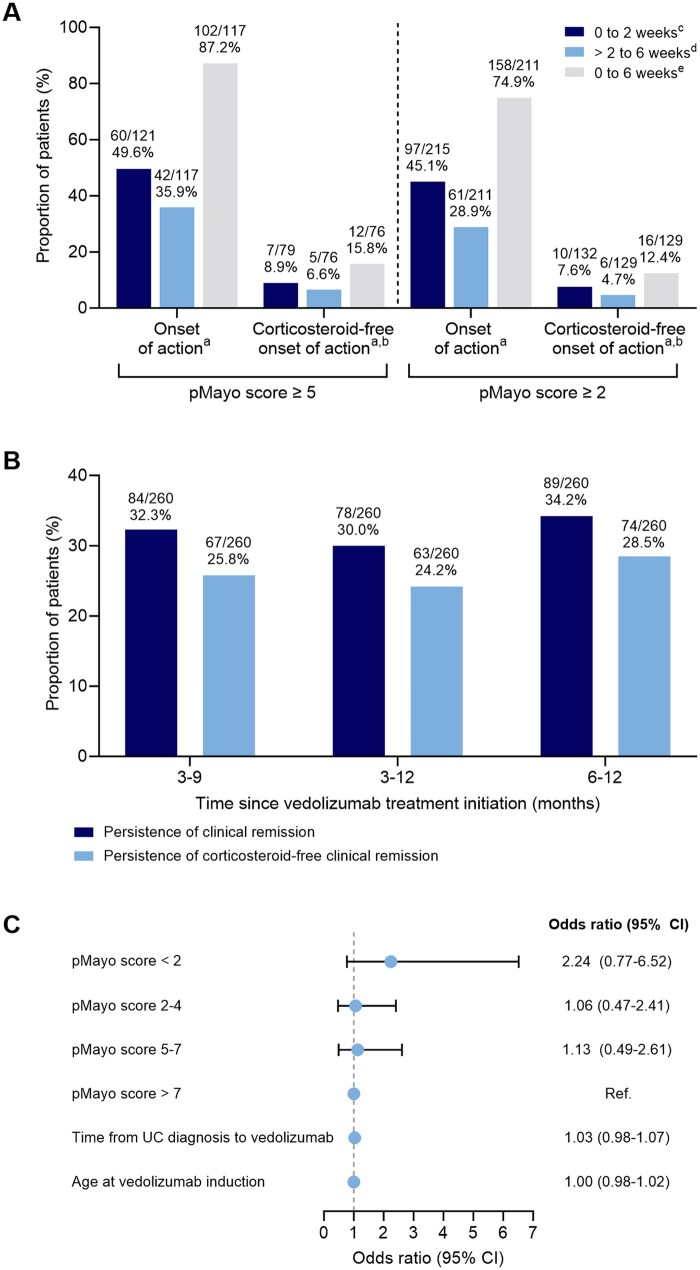
(A) Onset of action and corticosteroid-free onset of action for patients stratified by partial Mayo score, (B) persistence of clinical remission and corticosteroid-free clinical remission for all patients who received first-line vedolizumab, and (C) multivariate analysis of factors that influence the odds of treatment persistence at 12 months. ^a^Partial Mayo score decrease of ≥ 1 point in rectal bleeding or ≥ 1 point in stool frequency from baseline assessed at week 2 or week 6. ^b^Patients who are not receiving a concurrent corticosteroid at each time point. ^c^Patients with onset of action at week 2. Denominator excludes patients who discontinued or were lost to follow-up. ^d^Patients without onset of action at week 2 and with onset of action at week 6. Denominator excludes patients who discontinued or were lost to follow-up. ^e^All patients with onset of action by week 6. Denominator excludes patients who discontinued or were lost to follow-up at week 6. UC, ulcerative colitis.

In a further exploratory analysis, among patients with pMayo score ≥ 5 (moderate or severe disease) at baseline (excluding those in remission at baseline and discontinuations), 102/117 patients (87.2%) had onset of vedolizumab action by the week 6 assessment time point. This comprised 60 patients who had onset of action by week 2 and a further 42 patients who had onset of action between week 2 and week 6. When including patients with pMayo score ≥ 2 (mild, moderate, or severe disease) in the analysis, 158/211 patients (74.9%) had onset of action by the time of the week 6 assessment. The proportions of patients who had corticosteroid-free onset of action by week 6 were 15.8% for the pMayo score ≥ 5 and 12.4% for the pMayo score ≥ 2 subgroups, respectively (Figure 4A).

Persistence of clinical remission and persistence of corticosteroid-free clinical remission with first-line vedolizumab, which reflect the duration that patients stayed in remission, are shown in Figure 4B. Of all patients who started vedolizumab as a first-line biologic, 34.2% had remission between 6 and 12 months after initiation. Twelve months after beginning treatment with vedolizumab, 188/260 patients (72.3%) remained on treatment, and 72 patients had discontinued treatment. In a multivariate analysis of treatment persistence, none of the evaluated variables had a significant effect on the likelihood of patients remaining on treatment over time (Figure 4C).

## Discussion

This study provides real-world data showing that anti-TNFα treatments can be used successfully as second-line biologics without loss of effectiveness in patients with UC who have previously received vedolizumab. Twelve months after initiating a second-line anti-TNFα treatment, the proportions of patients who had a clinical response or clinical remission in the present study were similar to rates reported in previous studies of anti-TNFα treatment.^4^^,^^5^

Although biologics have demonstrated efficacy in treating patients with UC,^4–6^^,^^13^ the choice of which agent to use as the first-line biologic treatment remains unclear.^20^ Anti-TNFα treatments remain the most commonly used first-line biologic treatment, but there is evidence that biologics with different mechanisms of action, including vedolizumab, are more clinically effective first-line treatments for patients with UC.^7–10^ When choosing a first-line biologic treatment, it is necessary to consider other factors beyond their comparative effectiveness as first-line treatments. For example, despite their effectiveness, some patients with UC have a primary nonresponse or secondary loss of response to their first-line biologic treatment and may need to switch to a second-line biologic.^11^^,^^12^ It is important to understand how the treatment sequence may affect the effectiveness of a second-line treatment, should one be required.^20^ Evidence from the phase 3 GEMINI 1 and VARSITY studies shows higher rates of clinical response, clinical remission, and mucosal healing when vedolizumab is used as a first-line biologic in patients who are biologic-naive than when it is used as a second-line biologic following an anti-TNFα treatment.^8^^,^^13^

Taken together, vedolizumab’s effectiveness as a first-line biologic versus anti-TNFα treatments and diminished effectiveness when used after anti-TNFα treatments support its use as a first-line biologic in patients with UC. The present study provides new data that support this idea. First, in biologic-naive patients who received vedolizumab as a first-line biologic in our study, nearly three-quarters of patients remained on the drug for 12 months. Of these, approximately 80.0% had clinical remission, equating to 57.3% of the total population having remission at 12 months. These findings were in line with previously reported data. In a meta-analysis of seven observational studies and four interventional studies that included patients who received vedolizumab as a first-line biologic, the rates of clinical remission at week 52 in previously biologic-naive patients with UC were 63.9% and 54.0%, respectively.^14^ Second, among patients in our study who discontinued vedolizumab and received a second-line anti-TNFα treatment, 20.8% had clinical remission at 12 months. The rates of remission observed in patients who received an anti-TNFα treatment following vedolizumab are similar to those previously reported for first-line anti-TNFα treatment in biologic-naive patients.^4^^,^^5^ Importantly, although prior comparative data in UC are limited and there are differences between patient populations across studies, rates of clinical remission achieved with second-line anti-TNFα treatment following vedolizumab in the present study were like those previously reported in studies in which one anti-TNFα treatment was used after another (e.g. first-line infliximab and second-line adalimumab). In the VARSITY study, patients who had previously received an anti-TNFα treatment had clinical remission rates of 16.0% and 20.3% with second-line adalimumab and vedolizumab, respectively.^8^ Finally, a further finding from the present study that supports the use of vedolizumab as a first-line biologic is the onset of action within 6 weeks in most patients. Of 211 patients with pMayo score ≥ 2 at baseline (mild, moderate, or severe disease) who received first-line vedolizumab, 158 patients (74.9%) had onset of action within 6 weeks. Although direct comparative data are limited, a view among some clinicians that vedolizumab has slow onset of action relative to other biologics has gained traction. Our data suggest that the clinical benefit of first-line vedolizumab in patients with UC was manifest within the first 6 weeks after treatment initiation.

This study has some important limitations. The main limitation is that the number of patients we identified who received a second-line anti-TNFα treatment following discontinuation of vedolizumab is relatively small. The patients are also recruited from a limited geographical area, and we cannot rule out that environmental or other factors may have influenced the results. Patients were not randomized into the study, and therefore the population of patients who received vedolizumab as a first-line biologic may also be subject to some selection biases resulting from the treatment decisions made by the treating physicians. Finally, endoscopic data were not available, so response and remission were measured using the pMayo score, which relies on subjective patient-reported outcomes and physician assessment. This meant that the effectiveness of second-line anti-TNFα treatment following vedolizumab could not be evaluated using objective endoscopic endpoints, for example, mucosal healing.

## Conclusions

Consistent with clinical trials and real-world data, this study shows that vedolizumab is an effective treatment when used as a first-line biologic in biologic-naive patients with UC. The onset of action of vedolizumab is rapid with most patients having a response within 6 weeks. For patients who receive vedolizumab as a first-line biologic but subsequently require second-line treatment, the new data presented here supports the use of anti-TNFα treatments after vedolizumab without substantial loss of effectiveness. Our data show that anti-TNFα treatments can be used flexibly as first- and second-line treatments without major impacts on their effectiveness. However, a treatment sequence of vedolizumab as a first-line biologic followed by an anti-TNFα treatment may maximize the effectiveness of each line of therapy and deliver the optimal outcomes for patients with UC over their lifetime.

## Supplementary Material

otag042_Supplementary_Data

## Data Availability

The aggregated data that support the findings of this study are available on reasonable request from the corresponding author. The data are not publicly available owing to privacy or ethical restrictions.

## References

[1] Solitano V , D’Amico F, Zacharopoulou E, et al Early intervention in ulcerative colitis: ready for prime time? J Clin Med. 2020;9:2646. 10.3390/jcm9082646 32823997 PMC7464940

[2] Le Berre C , Ananthakrishnan AN, Danese S, et al Ulcerative colitis and Crohn’s disease have similar burden and goals for treatment. Clin Gastroenterol Hepatol. 2020;18:14-23. 10.1016/j.cgh.2019.07.005 31301452

[3] Feuerstein JD , Isaacs KL, Schneider Y, et al AGA Institute Clinical Guidelines Committee. AGA clinical practice guidelines on the management of moderate to severe ulcerative colitis. Gastroenterology. 2020;158:1450-1461. 10.1053/j.gastro.2020.01.006 31945371 PMC7175923

[4] Rutgeerts P , Sandborn WJ, Feagan BG, et al Infliximab for induction and maintenance therapy for ulcerative colitis. N Engl J Med. 2005;353:2462-2476. 10.1056/NEJMoa050516 16339095

[5] Sandborn WJ , van Assche G, Reinisch W, et al Adalimumab induces and maintains clinical remission in patients with moderate-to-severe ulcerative colitis. Gastroenterology. 2012;142:257-265. 10.1053/j.gastro.2011.10.032 22062358

[6] Feagan BG , Rutgeerts P, Sands BE, et al GEMINI 1 Study Group. Vedolizumab as induction and maintenance therapy for ulcerative colitis. N Engl J Med. 2013;369:699-710. 10.1056/NEJMoa1215734 23964932

[7] Lukin D , Faleck D, Xu R, et al Comparative safety and effectiveness of vedolizumab to tumor necrosis factor antagonist therapy for ulcerative colitis. Clin Gastroenterol Hepatol. 2022;20:126-135. 10.1016/j.cgh.2020.10.003 33039584 PMC8026779

[8] Sands BE , Peyrin-Biroulet L, Loftus EV Jr., et al VARSITY Study Group Vedolizumab versus adalimumab for moderate-to-severe ulcerative colitis. N Engl J Med. 2019;381:1215-1226. 10.1056/NEJMoa1905725 31553834

[9] Bokemeyer B , Plachta-Danielzik S, di Giuseppe R, et al Real-world effectiveness of vedolizumab compared to anti-TNF agents in biologic-naïve patients with ulcerative colitis: a two-year propensity-score-adjusted analysis from the prospective, observational VEDO(_IBD_) -study. Aliment Pharmacol Ther. 2023;58:429-442. 10.1111/apt.17616 37322825

[10] Sablich R , Urbano MT, Scarpa M, et al Vedolizumab is superior to infliximab in biologic naïve patients with ulcerative colitis. Sci Rep. 2023;13:1816. 10.1038/s41598-023-28907-3 36725872 PMC9892496

[11] Fine S , Papamichael K, Cheifetz AS. Etiology and management of lack or loss of response to anti-tumor necrosis factor therapy in patients with inflammatory bowel disease. Gastroenterol Hepatol (N Y). 2019;15:656-665. 31892912 PMC6935028

[12] Roda G , Jharap B, Neeraj N, et al Loss of response to anti-TNFs: definition, epidemiology, and management. Clin Transl Gastroenterol. 2016;7:e135. 10.1038/ctg.2015.63 26741065 PMC4737871

[13] Feagan BG , Rubin DT, Danese S, et al Efficacy of vedolizumab induction and maintenance therapy in patients with ulcerative colitis, regardless of prior exposure to tumor necrosis factor antagonists. Clin Gastroenterol Hepatol. 2017;15:229-239.e5. 10.1016/j.cgh.2016.08.044 27639327

[14] Attauabi M , Madsen GR, Bendtsen F, et al Vedolizumab as the first line of biologic therapy for ulcerative colitis and Crohn’s disease - a systematic review with meta-analysis. Dig Liver Dis. 2022;54:1168-1178. 10.1016/j.dld.2021.11.014 34903497

[15] Bouguen G , Nachury M, Nancey S, et al GETAID. Comparative efficacy of infliximab and vedolizumab after failure of a first anti-TNF in patients with ulcerative colitis: a double-blind randomized controlled trial (EFFICACI). J Crohns Colitis. 2025;19:i74-i74. 10.1093/ecco-jcc/jjae190.0038

[16] Na JE , Park YE, Park JH, et al Efficacy of second-line biological therapies in moderate to severe ulcerative colitis patients with prior failure of anti-tumor necrosis factor therapy: a multi-center study. J Pers Med. 2024;14:1066. 10.3390/jpm14101066 PMC11508867 39452572

[17] Noviello D , Savarino E, Fries W, et al Real-life effectiveness and safety of tofacitinib and vedolizumab as a second-line therapy in anti-TNFs experienced patients ulcerative colitis: Preliminary results of an IGIBD study (VE2TO-UC). J Crohns Colitis. 2024;18:i1650-i1651. 10.1093/ecco-jcc/jjad212.1038

[18] Bressler B , Yarur A, Silverberg MS, et al Vedolizumab and anti-tumour necrosis factor α real-world outcomes in biologic-naïve inflammatory bowel disease patients: Results from the EVOLVE study. J Crohns Colitis. 2021;15:1694-1706. 10.1093/ecco-jcc/jjab058 33786600 PMC8495488

[19] Ritter TE , Fourment C, Kuten SA, et al Second-line biologic therapy after vedolizumab. Am J Gastroenterol. 2019;114:S429-S430. 10.14309/01.ajg.0000592448.04563.6a

[20] Bressler B. Is there an optimal sequence of biologic therapies for inflammatory bowel disease? Ther Adv Gastroenterol. 2023;16:17562848231159452. 10.1177/17562848231159452 37057077 PMC10087655

