# Supplementary material for: A real-world observational study of second-line anti-TNFα treatment in patients with ulcerative colitis who received vedolizumab as a first-line biologic
Source: Crohns Colitis 360. 2026 May 15;8(2):otag042. doi: 10.1093/crocol/otag042 (PMC13221246; doi:10.1093/crocol/otag042)
Supplement: otag042_Supplementary_Data [file otag042_supplementary_data.docx]

**
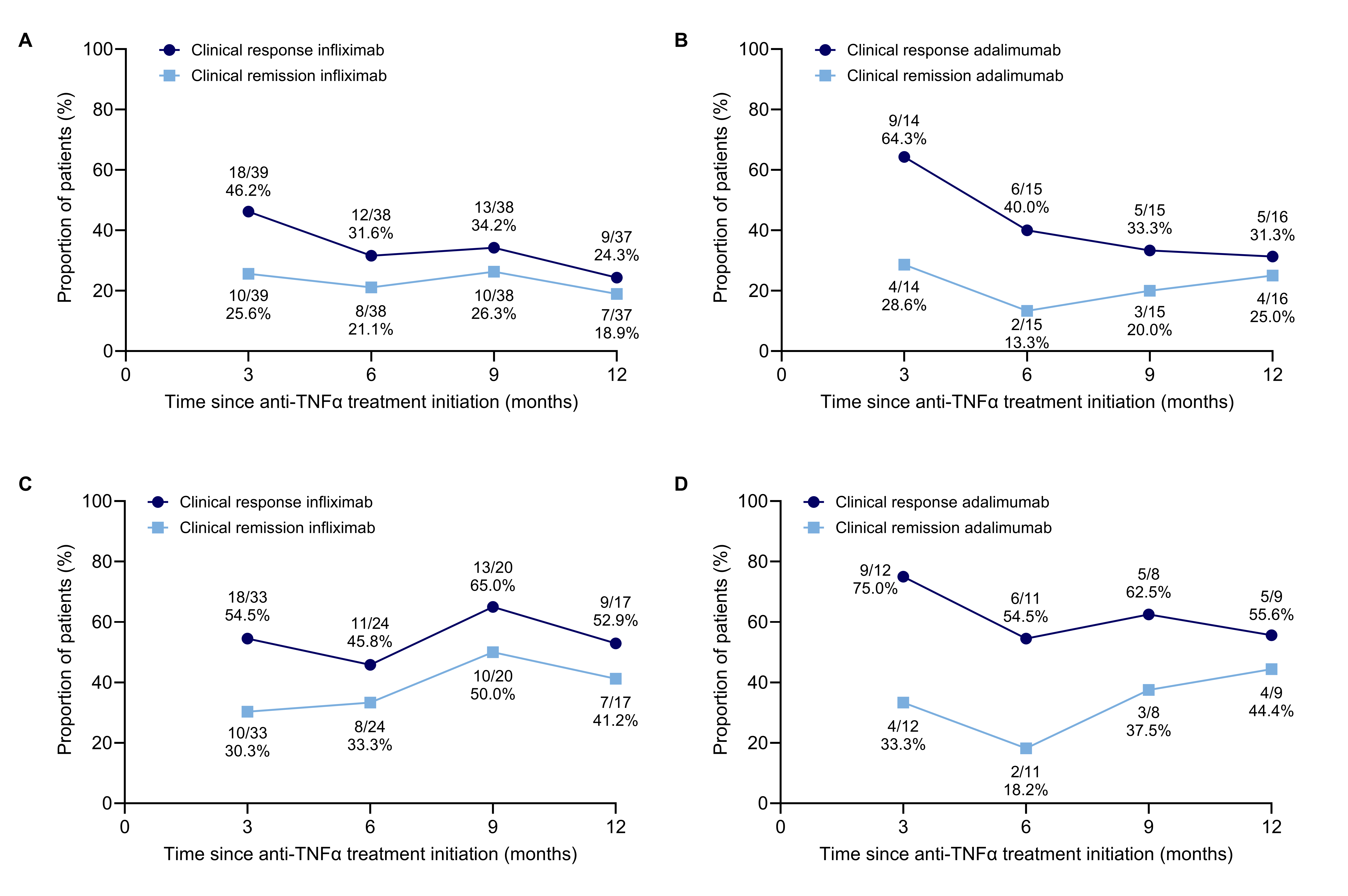
**

**Supplementary Figure 1.** Clinical response and clinical remission for all patients who received (A) second-line infliximab or (B) second-line adalimumab after previously receiving vedolizumab as a first-line biologic (intent to treat population) and clinical response and clinical remission for patients who remained on (C) second-line infliximab and (D) second-line adalimumab treatment at each assessment time point. TNF, tumor necrosis factor.
